# Aging alters across-hemisphere cortical dynamics during binaural temporal processing

**DOI:** 10.3389/fnins.2022.1060172

**Published:** 2023-01-10

**Authors:** Ann Clock Eddins, Erol J. Ozmeral, David A. Eddins

**Affiliations:** ^1^Department of Communication Sciences and Disorders, University of South Florida, Tampa, FL, United States; ^2^School of Communication Sciences and Disorders, University of Central Florida, Orlando, FL, United States

**Keywords:** electrophysiology, cortical auditory evoked potentials, hemispheric asymmetry, interaural time difference, binaural interaction component

## Abstract

Differences in the timing and intensity of sounds arriving at the two ears provide fundamental binaural cues that help us localize and segregate sounds in the environment. Neural encoding of these cues is commonly represented asymmetrically in the cortex with stronger activation in the hemisphere contralateral to the perceived spatial location. Although advancing age is known to degrade the perception of binaural cues, less is known about how the neural representation of such cues is impacted by age. Here, we use electroencephalography (EEG) to investigate age-related changes in the hemispheric distribution of interaural time difference (ITD) encoding based on cortical auditory evoked potentials (CAEPs) and derived binaural interaction component (BIC) measures in ten younger and ten older normal-hearing adults. Sensor-level analyses of the CAEP and BIC showed age-related differences in global field power, where older listeners had significantly larger responses than younger for both binaural metrics. Source-level analyses showed hemispheric differences in auditory cortex activity for left and right lateralized stimuli in younger adults, consistent with a contralateral activation model for processing ITDs. Older adults, however, showed reduced hemispheric asymmetry across ITDs, despite having overall larger responses than younger adults. Further, when averaged across ITD condition to evaluate changes in cortical asymmetry over time, there was a significant shift in laterality corresponding to the peak components (P1, N1, P2) in the source waveform that also was affected by age. These novel results demonstrate across-hemisphere cortical dynamics during binaural temporal processing that are altered with advancing age.

## 1. Introduction

Spatial hearing plays an important role in everyday activities such as driving in noisy traffic, crossing the street at an intersection, and listening to conversations in a crowded restaurant. Not surprisingly, converging evidence indicates that spatial hearing abilities in senescent listeners is impeded by degradations to the binaural auditory system (e.g., Dubno et al., 2008; Eddins and Hall, 2010; Ozmeral et al., 2016; Eddins and Eddins, 2018; Eddins et al., 2018; Gallun and Best, 2020), the key pathway for processing spatial auditory cues. While there is substantial interest in age-related changes in binaural processing and spatial hearing, the nature of those changes and their underlying mechanisms are not fully understood or characterized. Because the power in human communication (i.e., speech) and competing sounds is greatest at low frequencies, it is of value to understand the impact of aging on low-frequency dominant coding of binaural processes, such as coding of interaural time differences (ITD). In avian species, ITDs are topographically encoded *via* cellular arrays tuned to a narrow range of ITDs (Konishi, 2003), as suggested by Jeffress (1948). In mammals, however, converging research points toward a non-topographic, opponent-channel process in the cortex whereby ITDs are deduced from the relative neural activity of opposing channels broadly tuned to the midline and two spatial hemifields (Brand et al., 2002; McAlpine, 2005; Briley et al., 2013; Stecker et al., 2015; McLaughlin et al., 2016; Ozmeral et al., 2016, 2019). Moreover, when stimuli are presented or perceived from one hemifield versus the other, a majority of the cortical activity occurs in the contralateral hemisphere. Although somewhat modest, this contralateral bias has been demonstrated for ITD coding in humans based on both evoked potential (Salminen et al., 2009; Briley et al., 2013; Ozmeral et al., 2016) and functional magnetic resonance imaging (fMRI) BOLD measures (von Kriegstein et al., 2008; Gutschalk and Steinmann, 2015; Stecker et al., 2015; McLaughlin et al., 2016).

Binaural coding and contralateral bias may also be reflected in the binaural interaction component (BIC); a derived measure that can be computed from the auditory brainstem response (ABR), middle latency response (MLR), or the cortical auditory evoked potential (CAEP) (Dobie and Berlin, 1979; McPherson and Starr, 1993; Fowler and Horn, 2012; Van Yper et al., 2015; Dykstra et al., 2016; Laumen et al., 2016; Sammeth et al., 2020). The BIC is a difference waveform obtained by subtracting the algebraic sum of monaural responses to isolated left and right ear stimulation from the binaural response [B–(L + R)] or by computing the converse [(L + R)–B] (McPherson and Starr, 1993; Van Yper et al., 2015). Typically, the binaural response is smaller in amplitude than the summed monaural response giving rise to small difference components, or BIC, at different latencies depending on the measure being analyzed (i.e., ABR, MLR, CAEP). Although some studies have reported an inability to measure an acoustic BIC, even in normal-hearing subjects (Haywood et al., 2015), others suggest that it may serve as useful tool for binaural hearing tests (e.g., Riedel and Kollmeier, 2002; Benichoux et al., 2018; Brown et al., 2019). Further, electrically evoked BIC responses have been recorded in humans with bilateral cochlear implants (CI) (He et al., 2010; Gordon et al., 2012; Hu and Dietz, 2015; Hu et al., 2016) and in bilaterally implanted animals (e.g., in cat, Smith and Delgutte, 2007; Hancock et al., 2010).

The reduced amplitude of the binaural response is not well understood but may originate from a combination of contralateral inhibitory and ipsilateral excitatory neural populations in the superior olivary complex (SOC), as shown in data from cat (Ungan et al., 1997; Ungan and Yagcioglu, 2002) and guinea pig (Goksoy et al., 2005), and similarly modeled data (Gaumond and Psaltikidou, 1991). Such a population-based code for BIC generation is consistent with the population-based opponent channel model for ITD coding in the cortex (Magezi and Krumbholz, 2010; Salminen et al., 2010, 2015; Briley et al., 2013; Ozmeral et al., 2016) and thus may represent related underlying mechanisms of spatial processing. The BIC has been measured from both the ABR and MLR over a range of ITDs where it was shown to decrease in amplitude and increase in latency with increasing ITD (McPherson and Starr, 1995; Riedel and Kollmeier, 2006). Comparable BIC data for CAEPs in humans are limited (e.g., Henkin et al., 2015) and have not been reported across ITDs nor have they been used to assess contralateral bias.

The impact of advancing age on binaural coding in cortical evoked responses or BIC measures may reflect global age-related changes in sensory processing, such as reduced neural inhibition, specifically at the level of the SOC or higher (Willott et al., 1997; Caspary et al., 2008), or a more general reduction in temporal synchrony or increased temporal jitter (Pichora-Fuller et al., 2007; Ozmeral et al., 2016). Recent data indicate that reduced inhibition and reduced temporal synchrony both play a role in age-related changes in ITD processing that are stimulus or context dependent. That is, static or fixed ITDs with strong stimulus onset markers lead to larger evoked response amplitudes (i.e., reduced inhibition; Eddins et al., 2018) while dynamic shifts in ITD, following a different preceding ITD, result in smaller evoked response amplitudes (i.e., reduced temporal synchrony; Ozmeral et al., 2016) in older adults. In the present study we hypothesized that if binaural processing is influenced by a down-regulation in inhibition with age, then neural responses for all ITDs will be larger in older than in younger listeners for all ITDs. Alternatively, if reduced temporal synchrony is a primary age-related factor for binaural coding in older listeners, then neural responses for older listeners would be smaller than those for younger listeners, with the greatest difference occurring for large ITDs and smaller differences for ITDs approaching midline.

Importantly, aging can also influence the distribution of neural activity across the cortex such that it may alter the expected contralateral bias that occurs with ITD processing. In the context of cognitive aging, Cabeza (2002) proposed the hemispheric asymmetry reduction in older adults’ model (HAROLD) based on functional neuroimaging studies in which prefrontal activity during an episodic memory retrieval task was right lateralized in younger adults but showed bilateral activity in both hemispheres in older adults (Cabeza et al., 1997). One hypothesis for the reduced asymmetry is based on compensatory processes, whereby older adults recruit activity from other brain regions to compensate during increased task demands to help enhance performance. As a result, there is broader distribution of activity across hemispheres and reduced asymmetry toward one hemisphere or the other. A second hypothesis for decreased hemispheric lateralization with advancing age is based on the concept of functional dedifferentiation in which neural processes associated with cognitive strategies, and perhaps sensory processing specialization, become less organized or more distributed both regionally and globally across functional networks (Cabeza et al., 1997; Festini et al., 2018). Although development of the HAROLD model was based on asymmetry reductions in prefrontal cortex during cognitive tasks (e.g., episodic and working memory), additional data on visuospatial processing also suggests reduced hemispheric lateralization in older adults (e.g., Learmonth et al., 2017). It remains uncertain whether the model is generalizable to auditory sensory processes, such as ITD coding, that are known to elicit hemispheric bias in neural activation across the cortex. The present study thus serves as an ideal test case of the generality of the HAROLD model. As such, we test the hypotheses that advancing age alters neural encoding of ITD cues and contralateral bias, as indexed by both CAEP and BIC measures, and that such changes follow the HAROLD model whereby hemispheric asymmetry is reduced during sensory processing in older relative to younger listeners with normal hearing.

## 2. Materials and methods

### 2.1. Participants

**FIGURE 1:**
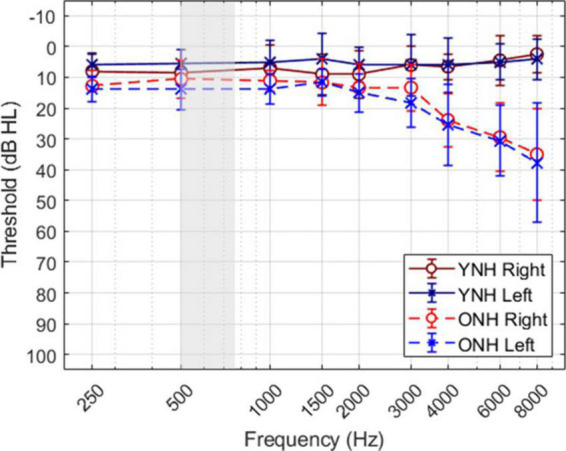
Mean and standard deviation of audiometric thresholds for each ear of each group.

A total of twenty individuals participated in the study; ten younger listeners (mean age ± SD, 24.9 ± 2.5 years; 9 females) and ten older listeners (70.0 ± 2.7 years; 6 females). The sample size was based on a power analysis with an effect size of 0.25, statistical power of 0.95, and alpha of 0.05. Figure 1 shows the mean and standard deviation of audiometric thresholds for both listener groups, where younger listeners (YNH) had clinically normal pure-tone thresholds ≤25 dB HL at octave frequencies from 250 to 8,000 Hz, and older listeners (ONH) had clinically-normal pure-tone thresholds ≤25 dB HL at octave frequencies from 250 to 4,000 Hz and ≤60 dB above 4,000 Hz. The gray shaded region illustrates the frequency bandwidth of the stimuli used in this study, as described below. The average threshold at 500 Hz (the frequency of focus in this study) across the two ears was 7 dB HL (±4.25) for the YNH group and 12.75 dB HL (±6.29) for the ONH group. All listeners were administered the Montreal Cognitive Assessment (MoCA; Nasreddine et al., 2005) to screen for cognitive impairment and all passed the screening with scores greater than 26. Each participant provided written consent and received hourly compensation for their participation, as approved by the University of South Florida Institutional Review Board.

### 2.2. Stimuli

Stimuli were band-pass Gaussian noise bursts, with lower and upper cutoffs of 500 and 750 Hz. Digital filtering was performed in the frequency domain using MATLAB^®^ (ver. R2018b, The Mathworks, Inc.). A new stimulus token was generated on each trial (sampling rate 24,414 Hz) with a duration of 400-ms, including 10-ms cosine-gated onset and offset ramps, and an inter stimulus interval of 1,600-ms. Stimuli were presented at a fixed level of 80 dB SPL *via* Tucker-Davis Technologies (TDT) RZ6 real-time processor, headphone buffer (HB7) and Etymotic ER-2 insert earphones. The stimuli were calibrated at the output of the earphones using a calibrator (B&K 4230), ear simulator (Knowles Electronics DB-100), 1/2” pressure microphone (B&K 4134), pre-amplifier (B&K 4134), and power conditioner (G.R.A.S. 12AA) routed to a multi-meter (Fluke 45).

Five binaural and two monaural stimulus conditions were run in block format. Binaural conditions included a diotic condition (i.e., ITD = 0 μs), and two left and two right leading ITDs at ±250 μs and ±500 μs (negative to the left, positive to the right). Due to sampling, true ITDs were 246 and 492 μs for the 250 and 500 μs conditions, respectively. Going forward, the conditions are referred to as L500, L250, Zero, R250, R500, with the letter corresponding to left (L) or right (R) leading, and number corresponding to the ITD value in μs. Monaural conditions included both left and right ear presentations. Each recording block consisted of 150 trials and lasted roughly 5 to 6 min, or total of about 45 min per subject with breaks given as needed. During each block, participants listened passively to the stimuli and were instructed to limit eye blinks and body movements while watching a captioned video of their own choosing. The video was used as a perceptual distractor during passive listening, as it has been shown to reduce movement artifacts and neural noise while not degrading response amplitudes or latencies (Pettigrew et al., 2004; Lavoie et al., 2008).

### 2.3. EEG data acquisition

Continuous electroencephalographic (EEG) responses were recorded using an ANT (Advanced Neuro-Technology BV) high-speed amplifier and an active shield, WaveGuard cap with 64 sintered Ag/AgCl electrodes (International 10–20 electrode system). Four additional electrodes were placed at the outer canthus of each eye and on the supra and infra orbital ridges of the left eye to monitor eye movement and blink activity. Electrode impedance was maintained below 10 kΩ across all electrodes. The EEG was recorded at a sampling rate of 512 Hz with 24-bit resolution using asalab™ acquisition software (ANT). Stimulus generation, presentation and event triggering were controlled by custom MATLAB^®^ (ver. R2018b) software scripts paired with asalab™ using activeX controls.

### 2.4. EEG data processing

All EEG data were preprocessed using the software suite Brainstorm (ver. brainstorm3, Tadel et al., 2011) and included the following steps: band-pass filtering (even-order linear phase FIR filter, based on a Kaiser window design) between 0.1 and 100 Hz, notch-filtering at 60 Hz (2nd order IIR notch filter with zero-phase lag, 2 Hz, 3-dB notch bandwidth), artifact detection to identify eye blinks, physical movement, and other extraneous activity (>150 μV), artifact removal *via* principal component analysis (PCA) and signal-space-projection (SSP), detrending to remove the DC signal, baseline correction (−100 to 0 ms), and re-referencing to the average across electrodes. Reponses were then epoched relative to stimulus onset (−200 to 600 ms). For sensor-level processing, epoched responses were averaged across trials (∼120 per condition) for each subject and each condition, and global-field power (GFP; Skrandies, 1990) was computed across electrodes. To evaluate peak components of the CAEP, GFP maxima were obtained within predefined temporal windows corresponding to the following components: P1, 40–70 ms; N1, 80–130 ms; and P2, 160–240 ms. CAEP and GFP grand average waveforms were computed for each condition for listeners within each subject group.

**FIGURE 2:**
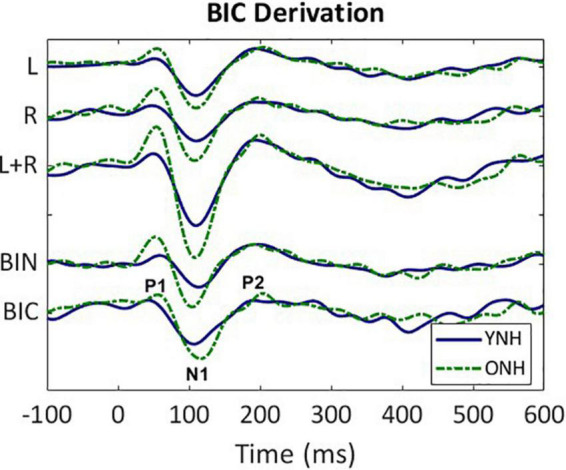
Derivation of the binaural interaction component (BIC). Grand average responses (arbitrary units) for each listener group (YNH–blue solid, ONH–green dashed); monaural left (L) and right (R) responses, sum of the monaural responses (L + R), binaural (BIN) response in the ITD Zero condition, and the BIC derived from the summed monaural (L + R) minus binaural (BIN) responses. The BIC is labeled with the three main peak components (P1, N1, P2).

The BIC responses were derived for all 64 sensors for each listener and each ITD condition. An exemplar of the derivation based on responses averaged across all listeners in the YNH (blue solid line) and ONH (green dashed line) groups for the ITD Zero condition is illustrated in Figure 2. The following steps were completed first for each subject before combining across subject group. First, the CAEP responses were averaged for each of the two monaural conditions (L, R) and were then added together (L + R). Next, the averaged binaural (BIN) response was subtracted from the summed monaural response to obtain the binaural interaction component [BIC = (R + L)–BIN]. These same steps were completed for each of the five ITD conditions. Like the CAEP peak quantification, BIC maxima were determined during the same temporal windows corresponding to peak components P1, N1, and P2.

### 2.5. EEG source localization analysis

Source localization analyses are designed to make use of scalp-based sensor responses from many electrodes to estimate underlying brain activity from potentially thousands of locations–the so-called inverse problem. Several computationally efficient source localization methods are available that derive brain activity from a linear recombination of sensor recordings. In this study, cortical sources from ongoing EEG responses were estimated using dynamic Statistical Parametric Mapping (dSPM; Dale et al., 2000) as implemented in Brainstorm. dSPM uses minimum norm estimation (MNE) methods to determine current density maps, and then normalizes the maps relative to estimates of the noise covariance in the responses to produce a z-score statistical map. The sources were constrained to the volume of the cortex and mapped to the Montreal Neurological Institute (MNI) Colin27 brain template (Holmes et al., 1998) using a multi-linear registration technique within Brainstorm. This approach uses the open-source software, OpenMEEG (Kybic et al., 2005; Gramfort et al., 2010) and forward models generated with the symmetric boundary element method (BEM). The cortical surface was parcellated into regions of interest (ROIs) defined in the Destrieux structural atlas (Destrieux et al., 2010). The auditory cortex was defined by three ROIs in left and right hemispheres that encompassed Heschl’s gyrus (HG, anterior transverse temporal gyrus), planum temporale (PT, temporal plane of the superior temporal gyrus), and the temporal sulcus (TS, transverse temporal sulcus). Similar to sensor-level analyses, source-localized waveforms were used to compute peak component maxima corresponding to P1, N1 and P2 for each listener and each condition. Likewise, source waveforms from monaural and binaural stimulus presentations were used to derive source-level BIC responses for each of the five ITD conditions.

### 2.6. Hemispheric asymmetry analysis

Using source-level data only, differences in hemispheric asymmetry with age and binaural condition for CAEP and BIC responses were quantified using a laterality index (LI) computed with the following equation:


LI=(|RH|-|LH|)/(|RH|+|LH|)

where, RH was the average response magnitude across the ROI sources in the right hemisphere and LH was the average magnitude of ROI sources in the left hemisphere. If LI = 0, then the magnitude of neural activity was essentially equivalent across hemispheres, whereas if LI > 0, dominant activity would be lateralized to the right hemisphere, and if LI < 0, dominant activity would be lateralized to the left hemisphere. The LI was computed based on the magnitude of the hemispheric activity averaged across peak components as well as separately for each peak component (P1, N1, P2).

### 2.7. Statistical analysis

Both sensor- and source-level data were used to evaluate changes in CAEP and BIC component amplitudes (P1, N1, P2) between age groups, across ITD conditions, and for source-level data only, across left and right hemispheres. Statistical analyses were completed on both sensor- and source-level data using SPSS (version 27). A mixed-design analysis of variance (ANOVA) was used to evaluate CAEP and BIC response amplitudes (P1, N1, P2) as a function of within-subject factors of condition (5 ITDs) and hemisphere (left, right), and between-subject factor of age group (YNH, ONH). Additional *post-hoc* analyses were completed as appropriate using pairwise comparisons with Bonferroni correction for multiple comparisons. To reduce Type I errors, all reported *F* values include degrees of freedom adjustments using Greenhouse-Geiser correction when significant deviations from sphericity were observed based on Mauchly’s test (Greenhouse and Geisser, 1959).

## 3. Results

### 3.1. Sensor-based measures of ITD processing: CAEP and BIC

**FIGURE 3:**
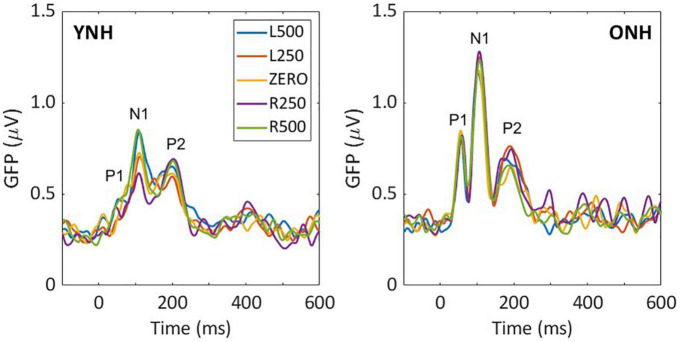
Grand average sensor-based global-field power (GFP) responses for each interaural time difference (ITD) condition and each listener group; young **(left)** and older **(right)**. The three main peak components are labeled in each panel (P1, N1, P2).

Low-frequency noise burst stimuli elicited transient neural responses with peaks corresponding to the P1-N1-P2 complex of the CAEP. The CAEPs from the 64 electrodes were used to compute the GFP for each listener and each stimulus condition. Figure 3 shows the grand average GFP across listeners for each group (YNH–left panel, ONH–right panel), for the five ITD conditions, with peak components labeled (P1-N1-P2). Two clear observations can be made when comparing responses across the two panels. First, older listeners demonstrated larger amplitude responses than younger listeners and little variation in amplitude across ITD conditions. Younger listeners, on the other hand, showed some amplitude variation with ITD conditions, most noticeably for N1, where the two extreme ITDs (L500, R500) produced the most robust responses. To quantify the observed differences, GFP amplitudes (combined across components P1, N1, P2) were submitted to a repeated-measures ANOVA to evaluate the between-subject factor of age group (YNH, ONH) and within-subject factor of ITD condition (L500, L250, Zero, R250, R500). The statistical results are reported in Table 1 and showed a significant main effect of age group [F(1,58) = 5.33, *p* = 0.025], supporting the observation that older listeners had larger responses overall than younger listeners. Second, despite the modest variation in response amplitude with changes in ITD for younger listeners, there was no significant main effect of ITD [F(4,232) = 1.13, *p* = 0.344] and no significant interaction between ITD and group [F(4,232) = 1.95, *p* = 0.113] on response amplitudes. GFP amplitudes were also evaluated independently for each peak component (P1, N1, P2) to assess the effects of age group and ITD condition. As reported in Table 1, repeated-measures ANOVA showed no significant main effect of age group on any of the three peak components (P1, N1, or P2), nor any main effect of ITD condition, and no significant interactions among the two factors.

**TABLE 1:** Repeated-measures analysis of variance (ANOVA) results for cortical auditory evoked potentials (CAEP) sensor-level measures.

	df	F	*p*
ITD	4, 232	1.129	0.342
Age	1, 58	**5.330**	**0.025**
ITD *×* Age	4, 232	1.950	0.113
**P1**			
ITD	4, 72	1.212	0.314
Age	1, 18	2.723	0.116
ITD *×* Age	4, 72	0.662	0.576
**N1**			
ITD	4, 72	0.488	0.700
Age	1, 18	0.609	0.445
ITD *×* Age	4, 72	0.423	0.745
**P2**			
ITD	4, 72	0.716	0.543
Age	1, 18	1.971	0.177
ITD × Age	4, 72	2.166	0.105

Significant F-statistic and corresponding *p*-values are shown in bold.

**FIGURE 4:**
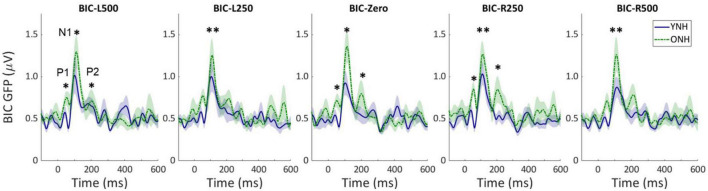
Grand average sensor-based global-field power (GFP) responses for the derived binaural interaction component (BIC) responses for each interaural time difference (ITD) condition and each listener group (YNH–blue solid, ONH–green dashed). The shaded region around each curve represents the ±1 standard error (SE) of the mean. The three main peak components are labeled in first panel (P1, N1, P2). Asterisks indicate significant group differences with the following *p*-values: *p < 0.05, ^**^p < *0.01*.

Similar to the CAEP results, the BIC derivation, as illustrated in Figure 2, showed that each response contributing to the derivation was larger in amplitude for the ONH than the YNH group, particularly for the summed monaural (L + R) responses. Replotting the BIC responses as GFP, Figure 4 shows a similar comparison between age groups across the five ITD conditions. In each condition, the mean responses for the ONH group (green dashed lines) consistently showed larger BIC amplitudes than the YNH group (blue solid lines). The shaded regions around each mean response function represents ±1 standard error of the mean (SEM). To evaluate the statistical significance of these observed differences, a repeated-measures ANOVA was completed to assess differences in the overall response amplitude (averaged across peak components) as well as differences for each peak component independently. As reported in Table 2, there was a significant main effect of age on overall response amplitude [F(1,58) = 19.629, *p* < 0.001], but no significant main effect of ITD condition [F(4,232) = 1.076, *p* = 0.369]. When evaluating effects of age and ITD condition on each peak component of the BIC, age had a significant effect on component amplitudes (see Table 2). *Post-hoc* pairwise comparisons, with Bonferroni correction for multiple comparisons, were completed to determine if differential effects of age might be observed for specific peak components across ITD conditions. As shown by the asterisks in Figure 4, significant age effects were observed for N1 across all five ITD conditions (L500, Zero, *p* < 0.05; L250, R250, R500, *p* < 0.01), whereas P1 and P2 showed significant age effects only for L500, Zero, and R250 (*p* < 0.05). Although age was shown to be a factor for sensor-based BIC amplitudes, ITD alone did not have a significant main effect on BIC response amplitudes nor was there any significant interaction between age and ITD condition.

**TABLE 2:** Repeated-measures analysis of variance (ANOVA) results for binaural interaction component (BIC) sensor-level measures.

	df	F	*p*
ITD	4, 232	1.076	0.369
Age	1, 58	**19.629**	**<0.001**
ITD *×* Age	4, 232	1.948	0.113
**P1**			
ITD	4, 72	1.192	0.322
Age	1, 18	**5.418**	**0.032**
ITD *×* Age	4, 72	0.707	0.590
**N1**			
ITD	4, 72	0.479	0.751
Age	1, 18	**8.774**	**0.008**
ITD *×* Age	4, 72	0.433	0.739
**P2**			
ITD	4, 72	0.713	0.586
Age	1, 18	**5.043**	**0.038**
ITD *×* Age	4, 72	2.172	0.081

Significant F-statistic and corresponding *p*-values are shown in bold.

### 3.2. Source-localized CAEP measures of ITD processing

A primary goal of this investigation was to evaluate potential age-related differences in hemispheric asymmetry during binaural processing. To do so, neural activity was quantified for source-localized responses derived from scalp-based responses using dSPM methods (Dale et al., 2000). Source responses were computed for three regions of interest (ROI) encompassing the primary auditory cortex in each hemisphere. Given that we did not obtain individual MRI scans from each participant but instead used the MNI Colin27 brain template along with the Destrieux atlas provided in Brainstorm, we chose to average responses across the three ROIs in each hemisphere and compute differences more broadly between left and right hemispheres (LH, RH).

**FIGURE 5:**
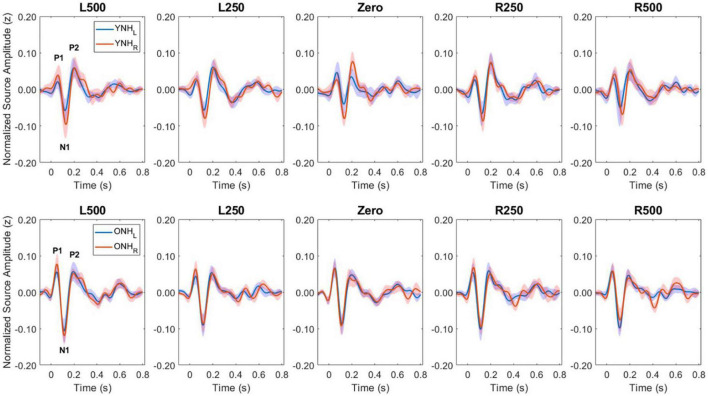
Grand average source-localized evoked responses from left (LH) and right (RH) hemisphere regions of interest (ROIs), for each interaural time difference (ITD) condition and each listener group (YNH, **top row**; ONH, **bottom row**). The three main peak components (P1, N1, P2) are labeled in first panel of each row.

Figure 5 illustrates the normalized source waveforms averaged across the three ROIs for left (blue lines and shading) and right (red lines and shading) hemisphere, with shading around each waveform corresponding to ±1 SEM. Responses are shown for younger (YNH–top panels) and older listeners (ONH–bottom panels) as a function of ITD condition. Unlike the sensor-based CAEP responses, the source-localized response amplitudes are more similar between the two groups and ITD conditions. A mixed model repeated-measures ANOVA was completed to evaluate differences in response amplitudes due to a between-subject factor of age group (YNH, ONH) and within-subject factors of ITD condition (L500, L250, Zero, R250, R500) and hemisphere (Left, Right). Analyses were completed based on amplitudes averaged across peak components (P1, N1, P2) and separately for each peak component. The results of those analyses are reported in Table 3.

**TABLE 3:** Repeated-measures analysis of variance (ANOVA) results for cortical auditory evoked potentials (CAEP) source-localized measures.

	df	F	*p*
ITD	4, 712	**7**.**826**	**<0**.**001**
Age	1, 178	0.591	0.443
Hemisphere	1, 178	2.960	0.087
ITD *×* Age	4, 712	**3**.**522**	**0**.**011**
ITD *×* Hemisphere	4, 712	**15**.**854**	**<0**.**001**
Hemisphere *×* Age	1, 178	0.602	0.439
ITD *×* Age *×* Hemisphere	4, 712	1.217	0.303
**P1**			
ITD	4, 232	0.367	0.832
Age	1, 58	**8**.**804**	**0**.**004**
Hemisphere	1, 58	1.378	0.245
ITD *×* Age	4, 232	**5**.**812**	**0**.**001**
ITD *×* Hemisphere	4, 232	**13**.**121**	**<0**.**001**
Hemisphere *×* Age	1, 58	0.153	0.697
ITD *×* Age *×* Hemisphere	4, 232	**4**.**071**	**0**.**003**
**N1**			
ITD	4, 232	**8**.**348**	**<0**.**001**
Age	1, 58	0.025	0.874
Hemisphere	1, 58	**13**.**548**	**0**.**001**
ITD *×* Age	4, 232	1.841	0.151
ITD *×* Hemisphere	4, 232	**13**.**175**	**<0**.**001**
Hemisphere *×* Age	1, 58	0.644	0.426
ITD *×* Age *×* Hemisphere	4, 232	**2**.**902**	**0**.**030**
**P2**			
ITD	4, 232	**3**.**080**	**0**.**023**
Age	1, 58	1.248	0.269
Hemisphere	1, 58	0.000	0.982
ITD *×* Age	4, 232	0.501	0.705
ITD *×* Hemisphere	4, 232	1.929	0.126
Hemisphere *×* Age	1, 58	0.801	0.375
ITD *×* Age *×* Hemisphere	4, 232	0.479	0.698

Significant F-statistic and corresponding *p*-values are shown in bold.

**FIGURE 6:**
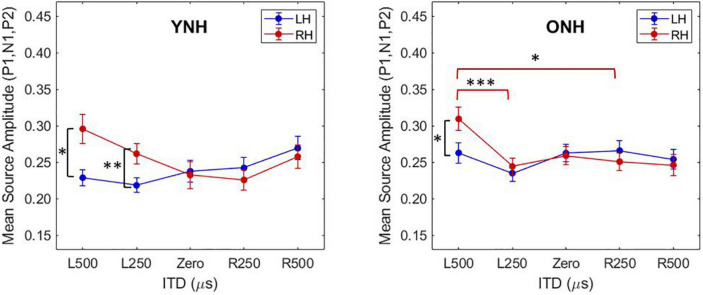
Mean source amplitudes averaged across absolute values of peak components (P1, N1, P2) in each hemisphere for each interaural time difference (ITD) condition and listener group (YNH, **left panel**; ONH, **right panel**). Significant differences between hemispheres were observed for both YNH (L500, L250) and ONH (L500) groups, and between conditions within right hemisphere only for ONH. Asterisks indicate significance levels: *p < 0.05, ^**^p < 0.01, ^***^p < *0.001*.

To better appreciate the overall differences in response magnitude between hemispheres for each ITD condition and each group, absolute values of the amplitudes for primary peak components (P1, N1, P2) were averaged and plotted by ITD, hemisphere and group, as shown in Figure 6. Consistent with the contralateral bias in binaural processing, young adults (left panel) showed greater right hemisphere activity for left leading ITDs (i.e., L500, L250) and greater left hemisphere activity for right leading ITDs (R500, R25), albeit somewhat smaller hemispheric bias for right leading stimuli. Older adults showed similar patterns, but smaller hemispheric differences across all ITDs. As reported in Table 3, statistically significant results were observed for the main effect of ITD [F(4,712) = 7.826, *p* < 0.001], as well as significant interactions between ITD and age [F(4,712) = 3.522, *p* = 0.011] and notably, between ITD and hemisphere [F(4,712) = 15.854, *p* < 0.001]. *Post-hoc* pairwise comparisons with Bonferroni correction showed that significant hemispheric differences were present in the YNH group for ITD conditions of L500 (*p* < 0.05) and L250 (*p* < 0.01) but only for L500 (*p* < 0.05) in the ONH group (see Figure 6). Further, significant differences in the ONH group were present in the right hemisphere only between the ITD conditions of L500 and L250 (*p* < 0.01) and between L500 and R250 (*p* < 0.05). Although not illustrated in a graphic format but reported in Table 3, analyses completed for each peak component showed significant three-way interactions between ITD, age and hemisphere for both P1 [F(4,232) = 4.071, *p* = 0.003] and N1 [F(4,232) = 2.902, *p* = 0.030] components. These results indicate a relatively complex relationship regarding how ITD cues are processed between hemispheres, over time (i.e., latency-based peak components) across age groups.

**FIGURE 7:**
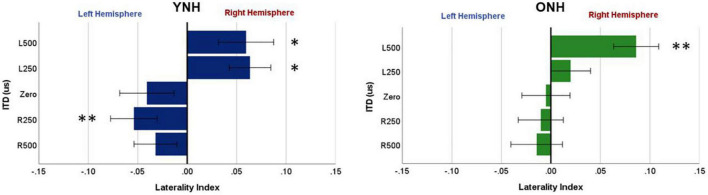
Laterality index quantified from source-localized left and right hemisphere regions of interest (ROIs) for each interaural time difference (ITD) condition averaged across each group (YNH, **left panel**; ONH, **right panel**). Significant laterality (relative to zero) for given ITD conditions is indicated for the following *p*-values: *p < *0.05*, ^**^p < *0.01*.

To further examine these complexities, we evaluated contralateral bias with the laterality index (LI) measure. The absolute values of peak component amplitudes in left and right hemisphere ROIs were averaged and used to compute the LI for each participant and each ITD condition. Figure 7 shows the mean LI results for YNH (left panel) and ONH (right panel) groups for each of the ITD conditions. Consistent with the contralateral bias model, left-leading ITDs produced greater lateralization toward the right hemisphere, whereas right-leading ITDs produced greater lateralization toward the left hemisphere. Statistical analyses based on a mixed model repeated measures ANOVA are reported in Table 4. The results showed that ITD had a significant effect on laterality [F(4,232) = 4.843, *p* = 0.002], but no significant differences were observed across age groups and no significant interactions between ITD and age were measured. Although both age groups demonstrated similar patterns across ITD, a one sample *t*-test was used to determine the extent to which each ITD condition produced significant asymmetry relative to zero. As indicated by the asterisks in Figure 7, the YNH group had statistically significant right lateralized activity for left-leading ITDs of L500 and L250 (*p < 0.05), and significant left lateralized activity for one right-leading ITD of R250 (^**^p < 0.01). The ONH group, on the other hand, only produced significant right hemisphere laterality for one left-leading L500 condition (^**^p < 0.01). These results are consistent with reduced hemispheric asymmetry in the older listeners during ITD processing.

**TABLE 4:** Repeated-measures analysis of variance (ANOVA) results for source-localized laterality index (LI) measures.

	df	F	*p*
ITD	4, 232	**4.843**	**0.002**
Age	1, 58	0.024	0.877
ITD *×* Age	4, 232	0.518	0.693
**P1**			
ITD	4, 72	**3.204**	**0.018**
Age	1, 18	0.142	0.711
ITD *×* Age	4, 72	1.598	0.184
**N1**			
ITD	4, 72	**3.270**	**0.016**
Age	1, 18	0.065	0.801
ITD *×* Age	4, 72	1.005	0.411
**P2**			
ITD	4, 72	1.235	0.304
Age	1, 18	0.005	0.945
ITD *×* Age	4, 72	0.380	0.822

Significant F-statistic and corresponding *p*-values are shown in bold.

**FIGURE 8:**
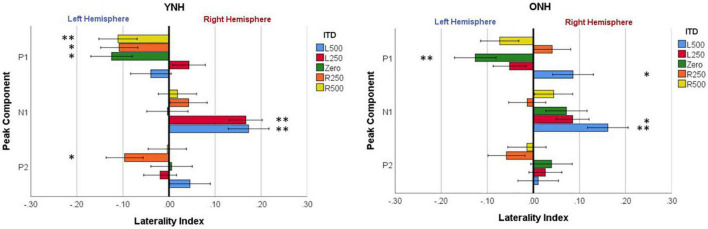
Mean laterality index for each interaural time difference (ITD) condition plotted relative to latency of peak components (P1, N1, P2) for YNH **(left panel)** and ONH **(right panel)** groups. Significant effect of peak component on laterality plotted relative to ITD condition is indicated for the following *p*-values: *p < 0.05, ^**^p < *0.01*.

**FIGURE 9:**
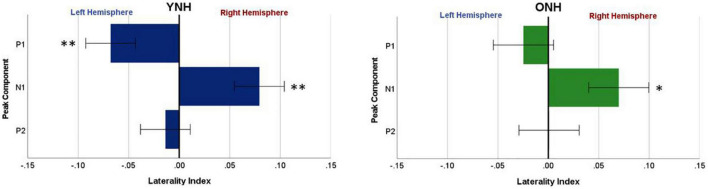
Mean laterality index, averaged across interaural time difference (ITD), plotted relative to latency of peak components (P1, N1, P2) for YNH **(left panel)** and ONH **(right panel)** groups. Significant laterality (relative to zero) for given components for each group separately is indicated for the following *p*-values: *p < 0.05, ^**^p < *0.01*.

To evaluate further the potential dynamic nature of lateralization over time, LI was quantified for each ITD condition in relation to the temporal sequence of CAEP peak components (P1, N1, P2). As indicated above, a one-sample *t*-test was used to determine which ITD conditions produced asymmetry relative zero for P1, N1, and P2 for each group. Figure 8 shows that some ITD conditions were lateralized differentially based on timing of the peak component for both YNH (left panel) and ONH (right panel) groups. For the YNH group, P1 was significantly left lateralized most notably for right-leading ITDs (R500, L250, Zero), whereas N1 was right lateralized for left-leading ITDs (L500, L250) and P2 shifted back to the left lateralization for one right-leading condition (R250). For the ONH group, significant laterality was observed in four ITD conditions across P1 and N1 peaks but was less orderly than the dynamic shifts observed for the YNH group. When averaged across ITD conditions, Figure 9 illustrates more directly how hemispheric laterality varied by timing of peak components. One-sample *t*-tests revealed that both groups had a similar dynamic pattern of left to right lateralization for P1 and N1, respectively, but the ONH group had reduced and non-significant P1 lateralization as compared to the YNH group. The statistical results of the repeated measures ANOVA evaluating laterality for each peak component for effects of ITD and age group are reported in Table 4. The results demonstrate a significant effect of ITD for P1 and N1 (as shown in Figure 8) but no significant effect of age group or interaction between age and ITD for any peak. Thus, based on laterality index measures, hemispheric dynamics during binaural temporal processing are influenced not only by the ITD stimulus condition but also by the time interval of the evoked response. Notably, the relationship between these factors is diminished in older relative to younger adults.

## 4. Discussion and conclusion

The overall goal of this project was to better understand the impact of advancing age on binaural processing and specifically on the encoding of binaural cues that support such processing. Here, we investigated the effects of advancing age on neural encoding of low-frequency ITD cues and the commonly observed contralateral bias in cortical processing, as indexed by both CAEP and BIC measures. The study design also allowed for assessment of the HAROLD hypothesis of reduced hemispheric asymmetry (or contralateral bias) as it might apply to auditory sensory processing in older adults during ITD processing.

### 4.1. Age-related changes in cortical processing of ITDs

Binaural processing, as evaluated for a range of different measures, is often degraded with advancing age (e.g., Dubno et al., 2008; Eddins and Hall, 2010; Ozmeral et al., 2016; Eddins and Eddins, 2018; Eddins et al., 2018; Gallun and Best, 2020). Although such degradation likely impacts everyday activities such as speech understanding in noisy backgrounds, the nature of such age-related processing changes and their underlying mechanisms are not fully understood or characterized (Gallun and Best, 2020). The present study was designed specifically to investigate the impact of aging on processing of low-frequency dominant ITD cues and their cortical representation in both younger and older normal-hearing listeners. Based on cortical responses to passively presented static ITDs, the results clearly demonstrated that grand average sensor-level CAEP responses were significantly larger for older than younger normal-hearing listeners across ITD conditions (see Figure 3 and Table 1). The responses for both groups, however, were not systematically altered by ITD. Additionally, when evaluating responses by peak component (P1, N1, P2), there were no systematic differences based on age or ITD condition (see Table 1).

The overall enhanced responses with age to static ITD stimuli are consistent with a down-regulation of inhibitory processing, as suggested by previous animal studies (e.g., Willott et al., 1997; Caspary et al., 2008) as well as human evoked potential studies of binaural processing (e.g., Eddins et al., 2018). This is in contrast to alternative age-related changes thought to result from reduced temporal synchrony (or increased temporal jitter), which would lead to smaller response amplitudes in older versus younger adults (e.g., Pichora-Fuller et al., 2007; Ozmeral et al., 2016). Amplitude reductions in the CAEP in older versus younger adults were observed in previous studies in response to dynamic changes in consecutive ITDs (Ozmeral et al., 2016). These changes were attributed to reduced temporal synchrony in the older group. Likewise, age-related reductions in CAEP amplitudes in older versus younger adults also have been observed during selective attention to spatial changes in sound location using comparable low-frequency ITD stimulus conditions (Ozmeral et al., 2021). Thus, the impact of age on whether CAEP amplitudes are enhanced or reduced appears to be more related to the nature of the stimulus presentation (static versus dynamic) and task demands (passive versus attention) rather than the attributes of the binaural stimulus *per se* (i.e., ITD).

To determine if other neural measures of binaural processing might shed more light on potential age-related differences in underlying function, the BIC was derived from sensor-level CAEP measures for each ITD condition. Like the CAEP analyses, the BIC results (when averaged across peak components) also demonstrated significant amplitude differences between age groups, where older listeners revealed significantly larger BIC responses across all ITD conditions (see Figure 4 and Table 2), yet ITD itself did not produce differences in amplitude for either group nor was there a significant interaction between ITD and age group. When BIC responses were analyzed independent by peak component (P1, N1, P2), the main effect of age was equally robust and significant, whereas ITD did not produce differences in amplitude nor were there significant interactions between ITD and age group for any of the peak components. To our knowledge, no previous studies of CAEP-based BIC measures with changes in ITD have been reported. Studies investigating ABR- and MLR-based BIC measures over a range ITDs, however, have shown mixed results in terms of how BIC responses change with ITD. Some studies have reported decreased BIC amplitudes and increased latencies with increasing ITD (McPherson and Starr, 1995; Riedel and Kollmeier, 2006), while a more recent normative study of ABR BIC showed no significant change in amplitude with ITD and substantial variability across a group of 40 young to middle-age normal-hearing participants (Sammeth et al., 2020). Differences in results between the present CAEP-based BIC responses and those from ABR- and MLR-based BIC measures are not surprising given the differences in the location of anatomical generators and additional contributors along the auditory pathway. Cortical measures in the present study likely have their origin in the brainstem, but they may reflect a decrease in temporal precision of the onset response to ITDs due to additional synaptic connections between the brainstem and cortex. The reduced temporal precision in CAEP compared to ABR or MLR BIC measures may then lead to smaller amplitude differences between ITD conditions. Nonetheless, both sensor-level CAEP and BIC analyses in the present study clearly demonstrate that advancing age leads to enhanced cortical responses to low-frequency ITD stimuli when presented in a passive, static mode with no systematic response variation across ITD conditions.

### 4.2. Age-related changes in hemispheric asymmetry during ITD processing

An important focus of this study was to test the hypothesis that advancing age in adults with normal hearing based on pure-tone thresholds leads to measurable changes in the expected contralateral bias in hemispheric processing of ITD cues often observed in studies of binaural processing (von Kriegstein et al., 2008; Salminen et al., 2009; Briley et al., 2013; Gutschalk and Steinmann, 2015; Stecker et al., 2015; McLaughlin et al., 2016; Ozmeral et al., 2016). Based on source-localized responses quantified from ROIs encompassing the primary auditory cortex in each hemisphere, the results showed that indeed ITD cues did elicit contralateral bias with greater response magnitudes in right hemisphere for left-leading ITDs and in left hemisphere for right-leading ITDs (see Figures 5, 6). These results are consistent with previous studies that have assessed both interaural timing and level difference encoding in the cortex using different neuroimaging methodology (Stecker et al., 2015; McLaughlin et al., 2016). Statistical analyses of data from the present study indicate that not only was there a main effect of ITD on CAEP source response magnitudes, but significant interactions also were revealed between ITD and hemisphere as well as between ITD and age group (see Table 3). These results demonstrate that ITD processing varies across hemisphere and that cortical processing of ITDs is differentially impacted by age. Notably, this novel outcome shows that advancing age leads to reduced hemispheric differences in source magnitudes when processing ITD cues (see Figure 6 and Table 3).

The most robust characterization of changes in hemispheric asymmetry was revealed with the laterality index analyses, as illustrated in Figures 7–9. First, both younger and older adults showed contralateral bias for some ITD conditions, as would be predicted from previous studies. Importantly, however, Figure 7 shows that younger listeners had significant laterality for both left- (L500, L250) and right-leading (R250) ITDs, whereas older listeners only showed significant laterality for one left-leading condition (L500). This novel demonstration of age effects on the laterality of cortical processing of ITDs is consistent with the HAROLD model such that older adults have reduced hemispheric asymmetry during binaural processing of ITD cues. Given that these stimuli were presented in a passive listening modality, it is less likely that the observed reduction in asymmetry results from compensatory mechanisms but instead may be linked to dedifferentiation in cortical processing of ITDs. Further studies are warranted to confirm the mechanistic source(s) of this age-related change. In addition, although the average hearing thresholds for both groups were within about 5 dB HL of one another at the frequency region of interest (500 to 750 Hz), there were differences between groups for higher frequency thresholds (≥4,000 Hz). Thus, the minimal differences in hearing sensitivity at 500 Hz and the influence of slightly poorer hearing two octaves above (i.e., ≥4,000 Hz) in the older group cannot be ruled out as a contributing factor to the observed age-related differences. Future work with clinically-significant hearing loss in the frequency region of interest can more definitively establish the impacts of typical age-related hearing loss.

Another novel outcome from this investigation, also related to hemispheric asymmetry, was revealed when examining the laterality index by each peak component of the CAEP source-localized response. When evaluating laterality by both ITD condition and peak component (see Figure 8), the LI results clearly demonstrated that hemispheric laterality shifts dynamically over time relative to the latency of the peak component of the evoked response. That is, the early response corresponding to P1 (∼40–70 ms post-stimulus onset) was significantly lateralized toward the left hemisphere and was driven largely by right-leading stimuli (e.g., R500, R250), whereas laterality during N1 (∼80–130 ms post-stimulus onset) was lateralized toward the right hemisphere and driven primarily by left-leading ITDs (L500, L250). P2 (∼160–240 ms), on the other hand, showed significant lateralization for only one ITD condition (R250) and one group (YNH). These results support the possibility that contralateral bias in hemispheric processing of ITD cues may be hierarchically processed such that right hemifield stimuli are processed earlier than left hemifield stimuli. Furthermore, as illustrated in Figure 9, both age groups showed similar laterality patterns as a function of peak component latencies, albeit older listeners showed reduced and non-significant laterality corresponding to P1. Taken together, the overall laterality results provide robust evidence of an age-related reduction in hemispheric asymmetry during ITD processing that is dynamically influenced over the time frame of the cortical evoked response. The relevance of such a binaural temporal processing scheme within and across hemispheres warrants further exploration and evaluation.

## Data availability statement

The raw data supporting the conclusions of this article will be made available by the authors, without undue reservation.

## Ethics statement

The studies involving human participants were reviewed and approved by University of South Florida Institutional Review Board. The patients/participants provided their written informed consent to participate in this study.

## Author contributions

AE wrote the initial draft of the manuscript with editorial contributions from EO and DE. All authors contributed equally to the experimental design, implementation, data collection, analysis, and interpretation.
